# In defense of decentralized research data management

**DOI:** 10.1515/nf-2020-0037

**Published:** 2021-01-11

**Authors:** Michael Hanke, Franco Pestilli, Adina S. Wagner, Christopher J. Markiewicz, Jean-Baptiste Poline, Yaroslav O. Halchenko

**Affiliations:** Institute of Neuroscience and Medicine Brain & Behavior (INM-7), Research Center Jülich, Wilhelm-Johnen-Straße, 52425 Jülich, Germany; and Institute of Systems Neuroscience, Medical Faculty, Heinrich Heine University, 40225 Düsseldorf, Germany; Department of Psychology, The University of Texas at Austin, 108 E Dean Keeton St, Austin, TX 78712, TX, USA; Institute of Neuroscience and Medicine Brain & Behavior (INM-7), Research Center Jülich, Wilhelm-Johnen-Straße, 52425 Jülich, Germany; Department of Psychology, Stanford University, 450 Jane Stanford Way, Building 420, Stanford, CA 94305, CA, USA; McConnell Brain Imaging Centre, Faculty of Medicine, McGill University, 3801 University Street, Montreal, Quebec, H3A 2B4, Canada; Department of Psychological and Brain Sciences, Dartmouth College, 419 Moore Hall, Hinman Box 6207, Hanover, NH 03755, NH, USA

**Keywords:** BrainLife, Canadian Open Neuroscience Platform, DataLad, Interoperability, OpenNeuro

## Abstract

Decentralized research data management (dRDM) systems handle digital research objects across participating nodes without critically relying on central services. We present four perspectives in defense of dRDM, illustrating that, in contrast to centralized or federated research data management solutions, a dRDM system based on heterogeneous but interoperable components can offer a sustainable, resilient, inclusive, and adaptive infrastructure for scientific stakeholders: An individual scientist or laboratory, a research institute, a domain data archive or cloud computing platform, and a collaborative multisite consortium. All perspectives share the use of a common, self-contained, portable data structure as an abstraction from current technology and service choices. In conjunction, the four perspectives review how varying requirements of independent scientific stakeholders can be addressed by a scalable, uniform dRDM solution and present a working system as an exemplary implementation.

## Zusammenfassung

Dezentrale Forschungsdatenmanagement (dFDM) Systeme verwalten digitale Forschungsdaten mit mehreren Teilnehmern, ohne dabei von einem zentralen Service abhängig zu sein. Zur Verteidigung von dFDM präsentieren wir vier Perspektiven: Einzelne Wissenschaftler, Institutionen, Datenarchive, Analyse-Plattformen und Konsortien, die zeigen, dass heterogene, aber auf interoperablen Komponenten basierende dFDM Systeme, im Gegensatz zu zentralisierten oder föderierten Lösungen, eine nachhaltige, resiliente, offene und anpassungsfähige Infrastruktur für wissenschaftliche Interessensgruppen sein können. Allen ist die Verwendung einer einheitlichen, portablen Datenstruktur gemein, die als Abstraktion von aktuell verwendeten Technologien zum Einsatz kommt. Zusammengenommen zeigen diese Perspektiven beispielhaft anhand eines in der Praxis verwendeten Systems, wie vielfältige Anforderungen unterschiedlicher Interessengruppen durch eine skalierbare dFDM Lösung adressiert werden können.

## Introduction

Research data management (RDM) is an increasingly important topic for individual scientists, institutions, infrastructure providers, and large-scale research collaborators. This shift in attention is driven by ethical considerations, threats to the trustworthiness of research outputs, and the desire to maximize the impact of publicly funded research. Generic, large-scale storage and computing infrastructure has existed internationally for a considerable time. Yet, the apparent lack of fit for domain-specific or regionalized data exchange and publication use cases has motivated a large number of localized, domain-specific developments or deployments of RDM solutions. These emerging solutions address some of the immediate needs, in part motivated by the increasing enforcement of minimum RDM standards by funding agencies. Yet as of today, the lack of infrastructure allowing interoperability across RDM systems still limits the potential impact that the research data can make to science and society.

This problem can be addressed by establishing a network of interoperable but independently governed and funded services that jointly form a decentralized research data management system (dRDM). Such a system makes digital research objects available across a network of participating institutions and investigators for publication, query, retrieval, backup or archive, and collaborative evolution. Importantly, this is achieved without critically relying on central services, thereby offering a high level of resilience against any failure of individual network components, including technical errors, but also institutional failure like discontinued funding.

Two primary models of decentralization can be distinguished: (1) A *federation*, where a single technology is utilized across partner sites, to provide a homogeneous solution, and (2) *interoperability*, where multiple technologies are used across partner sites but integrated into a single but heterogeneous set of components. On the one hand, the federation model dramatically simplifies the technical challenges. Simplicity comes at a cost though, as it constrains all partner sites to the deployment and maintenance of a single (homogeneous) software solution that might be suboptimal for many partners; a “one-size-must-fit-all” problem that can limit the type of partners involved in the federation. On the other hand, the interoperability model allows decentralization based on a network of heterogeneous software solutions. Each participant site is free to employ the optimal, site-specific solution avoiding the challenges and limitations of a “one-size-must-fit-all” approach. Though in such a system the challenge is shifted to establishing effective interoperability between the different technologies employed.

**Figure 1:**
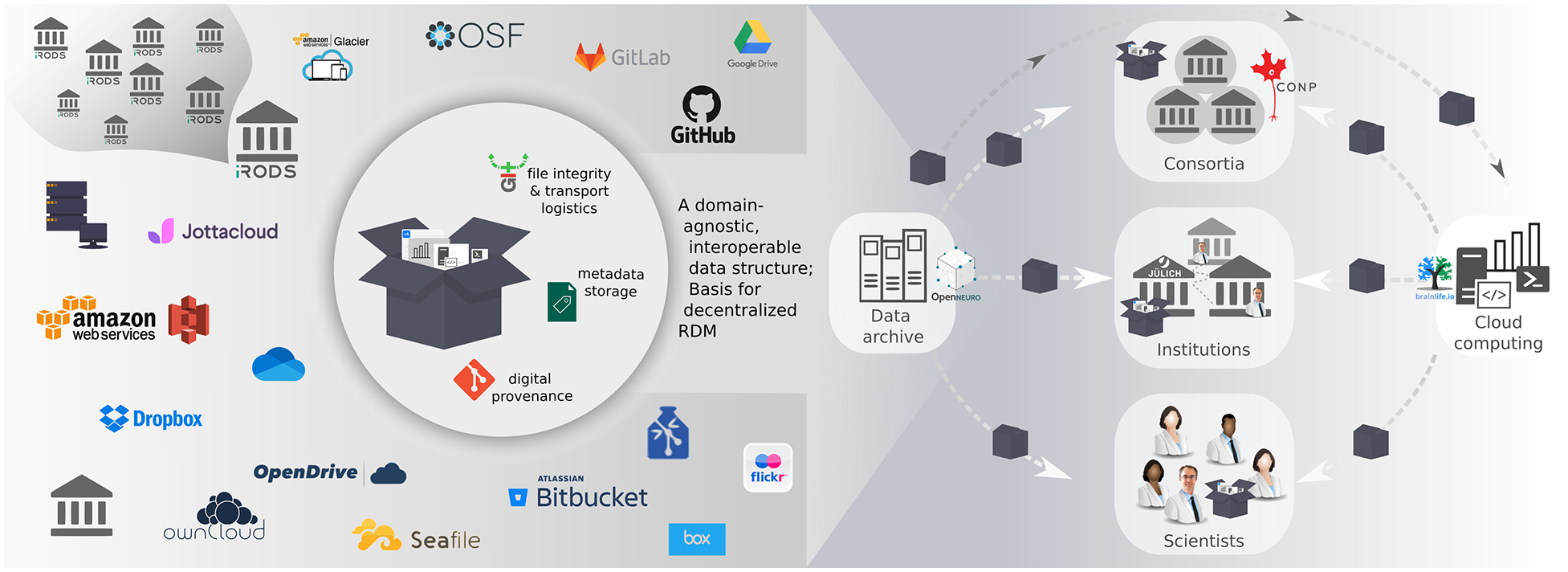
A common, portable data structure allows establishing interoperability between diverse participant sites. *Left:* A common data structure can serve as a uniform abstraction layer to interface any number of commercial or institutional storage services, which may be centralized or federated systems. *Right:* The portable nature of the data structure facilitates data exchange between archive and compute services, as well as collaboration among individual researchers or formal consortia. Moreover, it provides institutions with the flexibility to evolve their infrastructure without needlessly impacting scientific workflows.

Arguably, the interoperability model is more flexible and inclusive as it allows a more diverse set of partner sites to participate. More importantly, the interoperability model can improve the widespread application and resilience of dRDM. For example, established analysis and deployment workflows at each site can stay working, while interoperability with other sites can be established in parallel, for those projects requiring it, rather than requiring disruptive infrastructural changes that can simultaneously impact multiple laboratories or researchers. In the following, we present four perspectives on the utility of this type of dRDM. All four share a common principle: the use of a uniform data structure as a common denominator that facilitates independent development of software adapters to instruments and services that enable interoperability and data flow between all relevant infrastructure components and participants. While various standards and implementations of such data structures exist (e.g. BagIt, Kunze et al., 2018; Frictionless Data Package, Walsh et al., 2017; or Dat, McKelvey et al., 2020), all presented perspectives share the use of DataLad’s datasets (Hanke et al., 2020) as key technology choices. This particular implementation is a domain-agnostic lightweight data structure that offers joint version control capabilities for code and data (based on the industry standard Git, git-scm.com), supports arbitrarily structured metadata, and is capable of tracking the identity and availability of dataset components via the git-annex software (Hess, 2020) without requiring universal data access or actually containing the file content. This makes it possible to construct a dataset as a standardized overlay data structure which references content in heterogeneously organized data portals or databases. Moreover, it does not hide or bypass existing institutional access protection mechanisms and leaves authorization procedures in the responsibility of the data owners (see Figure 1).

## dRDM perspective: one laboratory or researcher

From the perspective of individual researchers, their laboratories, and collaborators, dRDM can improve day-to-day operations and make them robust against disruptive infrastructural changes. If data are uniformly accessible regardless of their storage location, scientists can orchestrate collaborative workflows and access not only to the data collected locally but also from external (public) resources in a streamlined fashion. Moreover, researchers utilizing a dRDM model can ensure consistent and robust data management across local and institutional information technology (IT) environments. For example dRDM makes it trivial to deploy a processing script from a local copy of data within the laboratory to a larger scale version of the data hosted in a datacenter. And as most researchers, in particular at early career stages, frequently move their workplace to different institutions (Guthrie et al., 2017), the benefits of this feature extend beyond a single workplace. When research agendas comprise a longer time frame, such that an employment change does not necessarily imply a fresh start and the discontinuation of previous projects, the potentially substantial and disruptive transition to a new institution and IT environment can be alleviated or prevented by a dRDM-based system.

Without dRDM, and depending on the magnitude of the differences between IT systems and policies, the necessary changes can be severe. Consider, for example, a transition from an environment with ample storage and shared computing resources, to a workplace with minimal local resources, but an institutional cloud storage service account. Before, all data holdings were accessible with low latency as if stored on a single big hard drive. Computing resources had direct data access, and analysis scripts could reference the desired data by (hardcoded) paths. After the transition, scripts cease to work because there is no local storage resource large enough to hold all data for analysis. Instead, additional, service-specific software has to be used to pull required data from the cloud and deposit results into the cloud. Essentially all analysis implementations of the past have to be manually adjusted to work in the new environment, an error-prone process that in itself is a threat to the reproducibility of results.

Using a common data structure as an abstraction of an analysis environment has the potential to substantially ease such transitions. In the case of a DataLad dataset, it is possible to comprehensively include all components of a compute- or data-intensive analysis in a single, version-controlled unit. This includes input data of any number and size, analysis code in any programming language, and even complete computational environments in the form of software container images. The dataset offers an intuitive application programming interface (API) for data access that hides the peculiarities of a particular IT environment and enables the development of analysis codes with improved portability properties. For example, a particular input file for an analysis can be referenced using a simple local path, relative to the root path of the analysis dataset: *input/datasetA/file1.dat*. An analysis script that requires this file can ensure this by executing the shell command *datalad get input/datasetA/file1.dat*. Importantly, the analysis script does not need to reflect that datasetA, which contains the file of interest, is a different modular data unit that is presently hosted on a particular storage service. Consequently, the analysis script does not need to be adjusted whenever the availability of datasetA changes because it has been transferred to a different institution. Instead, the DataLad software can be centrally configured to look for datasets, identified by a globally unique identifier and a precise version, at a different or additional location. Given that the data structure also allows for change tracking, it is possible to retrospectively discover how data were manipulated, improving the transparency and reproducibility of conducted projects.

For an individual researcher or laboratory, the barrier of entry into such a system is low. With no confinement to external services or file types, a scientist can transition new or existing projects into a common data structure independently and can typically achieve this without assistance, additional infrastructure, or project structure change. Nevertheless, the adoption of a common data structure such as DataLad’s datasets implies the necessity to acquire additional expertise, e.g. from documentation, user training, or tutorials, and also an individual’s interest in doing so. Efforts such as Repro-Nim’s (repronim.org) webinars, teaching resource collections, and teaching fellowships, or in-depth, user-focused documentation formats such as the DataLad Handbook (Wagner et al., 2020) facilitate this.

## dRDM perspective: a research institute

Like individual laboratories or researchers, research institutes also exist in a volatile environment. It is in their best interest to provide their scientists with the latest technologies to maximize their competitive advantage, boost research efficiency, and consequently increase the attractiveness and reputation of a research environment. However, the desire to quickly adopt new technologies has to be counterbalanced with the need to keep the cumulative cost of legacy infrastructure and procedures at a manageable level. This is compounded by the fact that institutions are generally responsible for guaranteeing a certain level of longevity for all research outputs, for example, the retention of research data, typically for at least a decade.

For the same reason as for individual researchers or laboratories, readiness for future infrastructure transitions, it makes sense for research institutions to utilize a portable, common data structure as an abstraction layer for RDM operations. The key feature of data structures, like DataLad’s datasets, is that they present researchers with a familiar view, a project directory on a filesystem, and internally translate *requests for data by location* (i.e. a file path) into *requests for data by identity* (i.e. a UUID or a checksum). This represents a powerful paradigm shift, as it enables future modifications of the content lookup and retrieval without changing the user/research-facing data representation.

The Institute of Neuroscience and Medicine Brain & Behaviour (INM-7) of the Research Center Jülich uses DataLad datasets not only to manage access to large-scale neuroimaging datasets, like the UKBiobank (Miller et al., 2016), or the Human Connectome Project (HCP, van Essen et al., 2013), but also as a system to archive completed projects. Institute members can discover all managed datasets via a collection that is maintained as a DataLad superdataset (a dataset comprising a versioned collection of datasets) hosted on a local GitLab (gitlab.com) instance. Independent of the hosting choice of the original data provider, institute members can access any data file by requesting it through the institute’s dataset collection, as described above. File access permissions are managed either directly by the respective data owners (e.g. each HCP user obtains their own credentials from the HCP consortium) or by controlled access to local downloads of restricted datasets (e.g. dedicated access group for signatories of the UKBiobank data usage agreement). Importantly, data access procedures remain uniform and fine-grained, regardless of whether an analysis is developed on a student’s laptop or is computed on the institute’s cluster system. This RDM setup also facilitates the *ad hoc* usage of resources at the Jülich Supercomputing Center (JSC). Institute staff can stage individual data resources on the JSC storage systems, and the DataLad software can transparently obtain dataset content on this independently operated resource without requiring individual adjustments of datasets, or analysis scripts. When a study is completed and archived, its DataLad dataset, including the incorporated study metadata, remains fully discoverable and accessible through the institute’s dataset collection. However, file content can be administratively moved from fast and expensive “hot” storage to higher latency bulk storage, and eventually onto tape backup systems, all without structurally impacting dataset access for institute members. Combined with data access statistics, this flexibility allows institute staff to maintain an optimal compromise of data access latency and storage demands without individual user negotiations.

## dRDM perspective: a domain data archive or computing platform

Domain data archives seek to provide high-reliability datasets access to all authorized researchers, with a secondary mandate to ensure that publicly funded data are findable via internal search or external indexing. Archives treat datasets as a natural unit of organization, and the necessary considerations are ingress, validation and metadata extraction, storage, publication, and egress. By adopting common data standards coupled with ingress and egress validation mechanisms, an archive team can focus development efforts on the key tasks of ensuring data access, availability, and findability.

For example, OpenNeuro (Gorgolewski et al., 2017) is a public neuroimaging data repository. Rather than imposing its own schema to which submitters must adapt their data, the archive adopted the community-developed Brain Imaging Data Structure (BIDS) standard for data organization and metadata (Gorgolewski et al., 2016). To assure reliable data access, and to serve the wide community of users, the archive relies on commercial infrastructure and uses Amazon Web Services to host the web interface and the Simple Storage Service (S3) to host the data. However, to ensure the long-term availability of the data, it requires a data model that is not tied to any specific vendor, hosting platform, or technology. In addition to the data model, OpenNeuro also desired making data available through generalized, stable interfaces independent of a particular storage platform or vendor. Consequently, the archive adopted DataLad to represent datasets internally (within the archive). This choice enables data change tracking and a common protocol for data egress (i.e. Git combined with git-annex). Data ingestion is also facilitated by DataLad. When a dataset is submitted to the archive, a DataLad dataset is created and binary files with imaging data are annexed. The dataset owner makes at least one “snapshot” to mark the dataset as complete and then publishes it in the archive. When the dataset is published, all files are uploaded to S3, and the URLs provided by S3 are associated with the annexed files. Finally, the DataLad dataset is published to a GitHub repository, to allow findability by other researchers even beyond the OpenNeuro Archive. The use of high-availability, permissive, third-party services ensures data are accessible even if the primary website suffers from downtime. At the same time, the data model does not depend on either service and can be ported to other services as new technologies emerge.

Version control and persistent identifiers are central features of the OpenNeuro data model. Datasets may change over time as new data are added or metadata is updated, and analyses of a dataset depend critically on the state of the dataset at the time of analysis. Dataset snapshots are represented as Git tags, allowing analyses to refer to the version of the dataset used via its version number (as opposed to by checksum). In addition, data object identifiers (DOIs) are issued for each snapshot of the dataset, ensuring that the particular version of the dataset may be cited in publications and facilitate the reproduction of analyses.

The use of DataLad and the published datasets on GitHub allows OpenNeuro datasets to be available beyond the archive. A variety of computational systems even without direct interaction with OpenNeuro can reference and access the datasets. For example, a researcher interested in developing a new analysis method might test the code during development on their personal computer by fetching an OpenNeuro dataset for testing or validation. The same researcher can then run a scaled-up version of the analysis on a high-performance computing cluster, which may host OpenNeuro datasets in a centralized location within a datacenter with minimal effort, simply reusing the data model and DataLad version tracking mechanisms. Finally, a cloud-based computational platform may expose OpenNeuro datasets to its users to increase data availability and enhance the general utility of the services offered.

As datasets are published and accumulate in one or several accessible repositories, new opportunities emerge for data aggregation and reuse across datasets (Avesani et al., 2019). Common metadata standards are essential to effectively harmonize data from multiple sources and enable research questions at scales previously impracticable. Furthermore, a common data standard can facilitate the aggregation of data from multiple sources. The effective separation of metadata (Git) and data (git-annex) is a key feature of the DataLad model that ensures that the metadata can be made accessible even when there are legal and ethical barriers to openly sharing data. It is thus becoming possible to develop tools to aggregate data from multiple providers without requiring an explicit effort from those providers. The dRDM model breaks some of the barriers and facilitates aggregation, curation, and upcycling data, allowing central archives such as OpenNeuro to act as stewards rather than gatekeepers.

Key partners that can be effectively served by the proposed dRDM model are cloud computing platforms. BrainLife (brainlife.io) is one of the most recent open and publicly funded platforms developed with the goal to serve researchers facilitating access, sharing, or reuse of data processing methods. The code implementing the data processing method can be submitted to BrainLife and registered as a web service (an App). The BrainLife platform allows automated tracking of the analyses execution and orchestrates data processing on diverse compute resources via a convenient graphical web interface or command line interfaces. BrainLife is not meant to be a data archive but a registry for reusable processing methods used in published scientific articles. The computational platform is compliant with the BIDS data standard so as to facilitate users’ data ingress and egress. Recently, the BrainLife team has used DataLad to connect the platform users with hundreds of BIDS-compliant datasets that are made publicly available as DataLad datasets. BrainLife uses DataLad to offer automated import “with the push of a button” of datasets that users have published on a variety of public archives. BrainLife benefits from the dRDM standardization in two ways: (1) Metadata standardization enables automatic identification of relevant dataset components, extraction of key data properties, and match-making of applicable analysis implementation against available data types, and (2) the abstraction of data transport logistics provided by DataLad’s datasets enables BrainLife to automatically obtain (pull) data files from the original providers, for example, from OpenNeuro, avoiding manual access to each data archive. Taken together, BrainLife is an example of a highly accessible computing platform that translates the potential of a dRDM system to the immediate computing needs of researchers, by connecting to independent standardization efforts without suffering from the need to continuously adjust to implementation changes in a large number of data portal and metadata access APIs.

## dRDM perspective: a collaborative multisite consortium, the Canadian Open Neuroscience Platform

The need for data sharing across institutions and states is fueled by the requirement of large sample sizes to enable well-powered and generalizable studies and for distributing the cost of data acquisition across sites. These large consortia generally opt for centralized data hosting, which simplifies data harmonization and management. However, large numbers can also be achieved through many independently acquired datasets that have the potential to better represent a more diverse population, an important factor for the construction of biomarkers. The Canadian Open Neuroscience Platform (CONP) is a consortium aiming for this goal and was funded in part to share neuroscience datasets across Canada within a comprehensive ethical and legal framework, establishing a repository of data implementing the Findable, Accessible, Interoperable, Reusable (FAIR) principles (Wilkinson et al., 2016).

While the central CONP data portal (portal.conp.ca) could have been only a set of links pointing to original infrastructures, this would not have given direct data access across datasets and would have been of limited utility for information aggregation. On the other extreme, centralizing data would have been infeasible. Critically, ethical or institutional policy requirements would have prevented transferring data to a central data storage for a number of datasets that are presently accessible on the platform. To keep the governance of datasets local, the CONP needed to adopt a distributed solution, while still making the data accessible directly through a single portal.

Adopting a portable, common data structure, like DataLad’s dataset, as an abstraction provided the CONP a shared and centralized space for distributing the metadata, while keeping the links to the original data locations. Metadata descriptors implemented using the DAta Tag Suite (DATS) model (Sansone et al., 2017) are incorporated in the centrally hosted dataset Git repositories, while original raw data are hosted on diverse platforms (OSF.io, Zenodo.org, Loris.ca, Braincode.ca, and others). The CONP uses a crawler to discover datasets on external services, like OSF or Zenodo, and builds a minimal DATS model for each dataset to make these data findable and accessible through the CONP portal. This offers a simple procedure for researchers who both want to share data in a general repository but also make these data discoverable in a neuroscience specialized portal.

Presently, CONP users must access datasets exclusively using the DataLad software. This imposes requirements, such as the necessity to deploy the software for any consumer. However, not all data consumption scenarios require that each participant operates a full-featured node of the dRDM system. Consequently, the CONP is working on convenient export functionality, such as an in-browser dataset downloader, to lower the technical threshold for interaction with its users. Because such a solution relies on standardized data access records, it can also be used by any other project using the data structure for dRDM.

## Conclusions

As illustrated by the four perspectives presented here, dRDM, built on a common, portable data structure that enables uniform access to all relevant commercial and institutional data services, is a flexible model that can scale from personal computing environments to individual institutions, all the way to large-scale collaborations in multisite consortia. The inclusive nature of this RDM approach that avoids one-size-must-fit-all prescription of centralized or federated services is suitable for introducing RDM standards and procedures in heterogeneous fields of endeavor. Consequently, it has also been selected as a strategic component of the NFDI Neuroscience initiative, a consortium that aims to consolidate neuroscience RDM in Germany along these lines.

Using the DataLad software and its datasets as an exemplary implementation of a common portable data structure, it is possible to curate and maintain unified data distributions collating data from the wide range of data providers. One such distribution is datasets.datalad.org, which currently provides a single point of entry for public or authenticated access to over 5,000 DataLad datasets covering over 200 TBs of neuroscience research data from hundreds of archives, initiatives, or individual laboratories. Among others, this collection also includes the superdatasets for CONP and OpenNeuro and through them provides access to all datasets managed by the respective entities. In turn, this collection is used by BrainLife to automatically discover datasets that can be processed on its platform.

Standardizing on a technology implies a substantial risk and installs a single point of failure in a complex system. However, standardization of core components also limits the variability that subsequent developments need to consider and ultimately enables more progress to be made with the same finite resources. In the case of DataLad, risks are introduced by three components: two small-scale developments (DataLad, git-annex) and the version control system Git. Git is a globally adopted industry standard. The chance of a technology failure without an adequate mitigation opportunity can be considered minimal. Both DataLad and git-annex build on Git, adding only documented, plain-text data structures to the content managed by Git. In the case of catastrophic failure (discontinuation of the development), the interpretability of data contained in these structures is unimpaired. Moreover, both software components are openly developed (public code history, issue tracker, support channels) and are available under recognized free software licenses (MIT, Affero GPL), such that continued maintenance by a third party can be considered feasible. This use of general-purpose protocols and technologies makes it possible to present scientific data in a readily usable form on platforms and forums, such as GitHub, that are used by a large audience of nonresearchers, thereby dramatically increasing the exposure of publicly funded research output, and successfully utilizes them for improving the capabilities and resilience of global dRDM.

## Glossary

API: An application programming interface (API) defines interactions between multiple software intermediaries. An API can be entirely custom, specific to a component, or it can be designed based on an industry standard to ensure interoperability.
Checksum: A checksum is a small-sized datum derived from a block of digital data for the purpose of detecting errors that may have been introduced during its transmission or storage.
UUID: A universally unique identifier (UUID) is a 128-bit number used to identify information in computer systems.
Version control: Version control (also known as revision control) is a class of systems responsible for managing changes to computer programs, documents, or other collections of information.
